# Professional certification for radiation therapists—adapting to a new language in MRI

**DOI:** 10.1002/jmrs.672

**Published:** 2023-03-21

**Authors:** Min Ku

**Affiliations:** ^1^ Australian Society of Medical Imaging and Radiation Therapy Melbourne Australia

## Abstract

Adoption and adaptation of new technology into a clinical environment requires a considered approach. The provision of appropriate education, training and credentialling of radiation therapists in the scope of magnetic resonance is of vital importance. Certification by the professional body is one mechanism to recognise this knowledge.

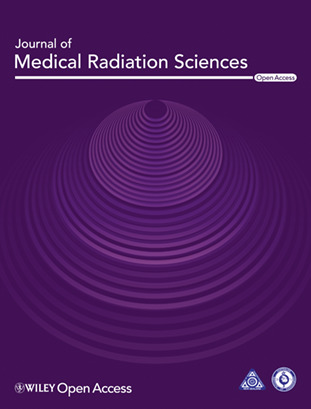

## Introduction

The Australian Society of Medical Imaging and Radiation Therapy (ASMIRT) is the peak body representing medical radiation practitioners in Australia. Our aims are to promote, encourage, cultivate and maintain the highest principles of practice and proficiency of medical radiation science.

With the introduction and implementation of Magnetic Resonance (MR) Linacs and MR Simulators (Sim) into Australian radiation therapy departments, it is incumbent upon ASMIRT to ensure safety and quality is maintained with this new delivery of service. MR Linacs are linear accelerators with integrated MRI capabilities for real time image acquisition and an MR Sim consists of an MRI platform which has been adapted for use in radiation therapy treatment planning.

ASMIRT acknowledges that provision of this service requires training, education and credentialling of radiation therapy (RT) staff in MR, in addition to leadership and guidance on adaptive treatment planning and verification processes. Radiation Therapy practitioners are part of a multidisciplinary team (MDT) responsible for accurate target delineation and treatment delivery. Current practice utilising MR Linacs has resulted in a change of role and scope of practice for radiation therapists and their MDT.^1^, ^2^

This special issue of Journal of Medical Radiation Sciences discusses the educational needs of the profession as it adopts and adapts to this new technology.

## Background

Radiation therapy has seen many changes in technology over the last century since the discovery of xrays.^3^ The evolution of technological improvements has seen the improvement in both planning and treatment applications enabling better healthcare outcomes for patients. The development of the linear accelerator, beam modification through the use of intensity modulated radiation therapy, and improved visualisation of tumours in three dimensions through the use of CT simulation provides efficiency and accuracy in target visualisation and delineation.^4^

As the quest for improved patient outcomes continues, the natural progression in image guidance is the use of MR. The use of MR technology for the purposes of radiation therapy and planning provides the ability to visualise anatomical changes in the live environment.^1^ The ability to make modifications and adapt patients' treatment with this accuracy has altered both the patient experience and enhanced professional capabilities.

## Education, Training and Credentialling

Currently ASMIRT offers a broad range of professional certification options. These certifications are not an academic qualification like a master's degree which has no clinical requirement, but rather a program that ensures a level of knowledge that is measurable and common across Australia. ASMIRT will be increasing this suite to include MR certification for radiation therapists. The current certifications consist of a theoretical examination and completion of clinical examinations and provides a nationally recognised benchmark for professional practice.

The original ASMIRT MRI Level 1 Certification for radiographers was created three decades ago to develop experience in a new and emerging technology. At that time, there was limited theoretical knowledge being taught in the pre‐undergraduate diploma era.

The creation of this certification pathway also provided opportunities for practitioners to expand their professional development without the need to pursue post graduate qualifications.

This editorial reviews the development of the ASMIRT MR in RT professional certification from inception to implementation.

The first MR linear accelerator (MR Linac) was installed in Australia in 2019^5^ creating the ability to facilitate online MR‐guided adaptive radiotherapy. The requirement for significant training and education has become apparent, as those intimately involved in the implementation of this technology will attest. Whilst radiation therapists are familiar with the MRI unit in the context of imaging for planning purposes, the depth of knowledge to appreciate the hardware, software and safety issues needed to be addressed. The concept of the MRI magnet being on all the time is very different to a linear accelerator where x‐rays can be controlled and turned on and off.

This highlighted that further education for radiation therapists in MRI was crucial. Furthermore, radiation therapists undertaking MRI are required to meet the Medical Radiation Practice Board of Australia's (MRPBAs) Professional capabilities for medical radiation practice Domain 1: Key Capabilities 8 & 9.2.^6^

## Partnerships

Whilst it is recognised that there is a plethora of information and resources readily available for learning about MRI, much of this is focussed on diagnostic applications. For departments that have implemented an MR Sim and/or MR Linac, there has been provision of vendor specific training which have led to individual departmental credentialing frameworks and workflows.^7^ It is evident from the literature that there needs to be consistency in training, credentialling, and exposure to a cross‐section of examinations of various complexity.^2^

In June 2020, the Queensland University of Technology (QUT) commenced an on‐line short course in MRI for radiation therapists.^8^, ^9^ As an extension of this program, a collaboration between QUT and ASMIRT was created to develop a novel certification to enhance the MR knowledge and skills of radiation therapists.

A Memorandum of Understanding was signed between the two organisations to construct an RT certification syllabus, resources and examination questions.

ASMIRT committee and reference group members, comprising MR experts and educationalists, were tasked with the development of a certification syllabus that would reflect the professional skillset of a radiation therapist performing MR imaging. The collaboration determined a consensus of syllabus content, and the subsequent development of the certification examination questions was undertaken in partnership with QUT.

## Pilot

To determine the standard of the content, a pilot examination for radiation therapist practitioners of varying clinical experience, from those a few years post undergraduate university graduation, and those that had undertaken vendor and other MRI knowledge‐based training was developed.

Organisations that have implemented MR Simulation and MR Linac technology were approached to advise of the pilot, and to seek volunteers to participate. A total of three organisations agreed to participate in the pilot held in November 2022 with their respective nominees. The syllabus and information regarding the online examination process was provided to the participants 3 months prior to the examination.

Participants were provided with detailed information regarding the requirements of the examination including familiarisation tests, supervisor and IT requirements. An examination week was allocated to accommodate both supervisors and participants. The online examination process was successfully completed, and a review is currently being undertaken of feedback provided by the participants.

It is anticipated that with these further improvements and the addition of a second part of the QUT MRI for Radiation Therapy online program focussing on the MR Linac, this ASMIRT professional certification will be rolled out fully in 2023. As this is an online examination process, overseas applicants will also be able to participate in this certification.

ASMIRT is providing this Level 1 certification as recognition that a radiation therapy (RT) medical radiation practitioner (MRP) is performing with professional skill in Magnetic Resonance (MR) imaging. This Level 1 certification describes capability at the level of an advanced beginner in the use of this novel RT technology. Further training and clinical experience are required to demonstrate expert practice. Patient care is a multidisciplinary approach and each organisation utilising this technology will adopt policies and procedures that best suit their own clinical environments. This Certification may be one mechanism to demonstrate MR knowledge.

## Conclusion

ASMIRT believes that the development of this certification will provide many learning opportunities for practitioners with an interest in MR and expanding their knowledge in this specific scope of practice. This professional certification will provide a benchmark of industry‐standard skill, and professional recognition for radiation therapy practitioners in the workplace, encouraging further development and growth in this rapidly expanding area.

## Funding

Nil.
